# 

**Published:** 1886-08-20

**Authors:** 


### TABLE OF CONTENTS

### Original and Selected Articles.

A Case of Suppressed Menstruation, by H. Christopher, M.D., of Missouri ....................285
A Case of Intussusception with Occlusion, by J. P. Motley, M. D , of Daviston, Ala ........... ..287
A Report Embodying two Hundred Cases of Tonsillitis, by J. M. G. Carter, M. D., Waukegan, Illinois....................288
Elevated Temperature in some Cases of Pernicious (Congestive) Ma'arial Fevers, by Jos. Jones, M.D., Louisiana....................292
Malarial Hæmaturia, by Charles C. Thornton, of Thornton, Holmes county, Miss....................294
Epilepsy, by C. L. Dana, M. D., of New York..297
Large Doses Ammonia Carbonate in the Suffocative Stages of Acute Pulmonary Diseases, by Benj. H. Riggs, M.D., Selma. Alabama......299
New Observations upon the Etiology of Eclampsia; Bismuth as a Dressing for Wounds.........301

### Abstracts and Gleanings.

The Treatment of Acute Sporadic Dysentery...302
The "Cent" Sign in Pleurisy ....................303
Anti-Rheumatic Mixture; Small Doses............304
Education and care of the Idiot....................305
The "Dead-Finger Symptom" in Bright's Disease; The Treatment of Compound Fractures by Wiring and Drainage.................... 306
Tuberculosis....................307
Permanent Drainage in Ascites; Case of Re-Injection of Blood during Amputation at the Hip-joint, with Rapid Recovery....................308
Antipyrin in Disordered Stomach; Early Removal of Sutures....................309
Hyperpyrexia in Rheumatic Fever; The Kawa Plant ....................310
Cubeb Inhalations in Diphtheria; Pelvic Anodyne; Total Extirpation of the Tongue by Kocher's Method....................311
Treatment of Severe Cases of Burning by Hebra's Water-bed; Morphine Subcutaneously in Infantile Convulsions; Deaths from Anæsthetics in 1885....................312
After Treatment of Cataract; Convulsions During Pregnancy Treated Successfully by Subcutaneous Injections of Pilocarpin; Ergot for Dysentery; The Editor's Work....................313
Treatment of Facial Neuralgia by Cocaine; Fluorides of Ammonia and Iron; Digitalis Treatment of Pneumonia....................314

### Scientific Items.

Struck by a Meteor; Cyclamose—a New Sugar; Artificial Cocaine....................315
New Light on the Germ Theory; A Few Remarks on Papayotin and Papain; The Chemical Barometer....................316

### Practical Notes and Formulæ.

Blackberry Cordial; Jamaica Dogwood; French Shoe Polish; Menthol in the Treatment of Urticaria; A Paste for Use in Eczema............317
An Application to Prevent Scarring after Variola; Elixir of Pepsin; Meaning of Spiritus Vini Gallici; Glycerin in Inhalation for Cough ....................318

### Editorials and Miscellaneous.

A Liberal Offer; Editorial Notices.................... 319
Antiseptic Cologne: Correspondence....................321
Books and Pamphlets Received.................... 322
Special Notices....................324

